# Air Pollution and Osteoporosis

**DOI:** 10.1007/s11914-024-00889-9

**Published:** 2024-09-20

**Authors:** Olivia Allen, Martin M. Knight, Stefaan W. Verbruggen

**Affiliations:** 1 Centre for Predictive in vitro Models, Queen Mary University of London, London, UK; 2 Centre for Bioengineering, School of Engineering and Materials Science, Queen Mary University of London, London, E1 4NS UK; 3 Digital Environment Research Institute, Queen Mary University of London, London, UK; 4 INSIGNEO Institute for in silico Medicine, University of Sheffield, Sheffield, UK

**Keywords:** Osteoporosis, Air pollution, Inflammation, Bone mineral density

## Abstract

**Purpose of Review:**

The purpose of this review is to provide a background of osteoporosis and air pollution, discussing increasing incidence of the disease with exposure to pollutants and the role that inflammation may play in this process.

**Recent Findings:**

Osteoporosis-related fractures are one of the most pressing challenges for the ageing global population, with significant increases in mortality known to occur after major osteoporotic fractures in the elderly population. Recent studies have established a firm correlative link between areas of high air pollution and increased risk of osteoporosis, particularly alarming given the increasingly urban global population. While the culprit pollutants and molecular mechanisms underlying this phenomenon have not yet been elucidated, initial studies suggest a role for inflammatory cascades in this phenomenon.

**Summary:**

While much more research is required to identify the most damaging air pollutants and to delineate the specific inflammatory molecular mechanisms, it is clear from the literature that shedding light on these pathways would unveil potential therapeutic targets to treat bone diseases, including osteoporosis. Major deficiencies of current animal models highlight the need for complex human *in* *vitro* models such as organ-on-a-chip technology to better understand the impact of air pollution.

## Introduction

This review begins with a brief discussion of the the key drivers of osteoporosis, and the current standard of care. This is then followed by a review of air pollutants, their influence on human disease and the role played by inflammation in these conditions. The article next explores the putative link between air pollution and osteoporosis, discussing the state-of-the-art in the field, before concluding with a future perspective on the potential of targeting air pollution and related inflammatory pathways to inhibit the development of osteoporosis.

## Osteoporosis

Osteoporosis presents as loss of bone mass, leading to fractures, severe pain, deformity and increased rates of mortality [1]. Clinically, the disease is classified as either primary or secondary osteoporosis. Primary osteoporosis refers to both bone loss occurring due to oestrogen deficiency in post-menopausal women (type I) and bone loss associated with the normal ageing process (type II). Secondary osteoporosis describes bone loss that occurs due to other diseases (e.g. cancer) or drug treatment (e.g. chemotherapies). Post-menopausal osteoporosis (type I), as the most common diagnosis, arises as the result of deficient oestrogen following the menopause [2].

Healthy bone maintains its strength and mineral homeostasis via bone remodelling, which is a coordinated and balanced process whereby osteoclasts continuously resorb aged or damaged bone and osteoblasts reform new bone tissue in its place [3]. However, this balance is perturbed during oestrogen deficiency, with osteoclasts removing excess bone without adequate formation by osteoblasts [4]. With the continuation of this process, bone loss manifests when trabeculae (internal supporting struts of bone) become thin and resorb completely, or fracture [5]. Eventually, this process allows debilitating bone fractures to occur under minimal trauma in the bones of the hip, wrist and spine.

The healthy remodelling process is also disrupted by disuse due to skeletal mechanical unloading [6]. A range of mechanosensing mechanisms exist in bone cells, such as mesenchymal stromal cells (MSCs) [7], including the primary cilium, a solitary sensory organelle that protrudes from the membrane of all bone cells that has been shown to act as key mediators of inflammatory signalling and mechanotransduction [8]. Mechanical stimulation via primary cilia, for instance by oscillatory fluid flow-induced shear stress, triggers osteogenic differentiation [9]. Primary cilium expression is similarly crucial in the process of osteoclastogenisis, with recent work demonstrating that increased primary cilium expression can inhibit osteoclast formation [10]. Furthermore, the primary cilium is well known to play an important role in mechanotransduction by osteocytes [11–13], thought to be the master orchestrator of bone adaptation to mechanical loading in health [14] and during osteoporosis [15, 16]. Thus, lack of mechanical stimulation can ultimately lead to imbalance of bone remodelling.

Age-related fractures are increasingly common. For example, in the US approximately ∼2.1 million osteoporosis-related bone fractures occur annually [17, 18]. Osteoporosis impacts women more than men, with 80% of the estimated 10 million Americans with osteoporosis being women. and one in two women over 50 experiencing a bone fracture because of osteoporosis [19]. Indeed, women over 45 years of age spend more days in hospital due to osteoporosis than diabetes, heart attack or breast cancer [20].

While a number of established diagnosis and treatment options exist for osteoporosis, clear deficiencies remain, highlighting the need for further research into treatment and prevention. Indeed, with a rapidly growing global population of ageing individuals, uncovering new mechanisms underlying the development of osteoporosis and ways to mitigate them is becoming increasingly urgent. Even more concerning, given the increasingly urban world population, air pollutants have recently been implicated in the development of osteoporosis, as will be discussed hereafter.

## Air Pollution

Air pollution has been highlighted as the greatest environmental threat to individual human health; according to the World Health Organization (WHO), 99% of the population breathe air that exceeds their guidelines on safe pollutant levels [21]. A 2019 study suggested that excessive levels of air pollution may be responsible for 8.79 million deaths per year globally [22]. Exposure to high levels of air pollution increases the risk of developing numerous diseases with significant effects on morbidity and mortality. Diseases currently linked to air pollution include cardiovascular disease [22], cancer [23], respiratory diseases [24], diabetes mellitus [25], immune disorders [26], and neurological disorders [27]. It is estimated that worldwide, each year seven million deaths can be attributed to the effects of ambient and household air pollution [21].

Air pollution is generally defined as solid, liquid and gaseous compounds that affect biological systems through one mechanism or another. Major sources of air pollution include vehicle emissions, industrial processes, power generation, and wildfires. Forms of air pollution can include gases such as ozone (O3), and noxious gases such as carbon dioxide (CO2), carbon monoxide (CO), nitrogen oxides (NO, NO2) and sulphur oxides (SO, SO2), as well as volatile organic compounds [28]. Pollution can also include particulate matter (PM), which can be classified according to the nature of particles, as biological, chemical, mineral and metal. However, while varied in nature, their inflammatory action is classified based on particle size, with diameter of PM ≤ 10 μm, ≤ 2.5 μm, ≤ 1 μm, ≤ 100 nm all classified as coarse particles (PM10), and in order of decreasing size fine particles (PM2.5), very fine particles (PM1.0) and ultrafine particles (PM0.1 or UFPs), respectively. Although the mechanism of air pollution affecting the lungs is obvious, how air pollutants can affect other body systems is still poorly understood and an area of broad study.

## Air Pollutants and Inflammation

The effects of air pollution on organs distant from the lungs, the site of inhalation, is thought to lead to health defects due to oxidative stress or inflammation [29]. While it is currently unclear which components of air pollution may trigger immune and inflammatory responses, and by what mechanism, there are multiple studies into the various types of pollutants and the diseases they are linked to.

Particulate matter, comprising extremely small particles, is able to enter the bloodstream via inhalation, and is known to trigger the systemic release of proinflammatory cytokines, including TNF-α, IL-1, IL-6 and IL-8 [26, 30, 31], and to elevate the incidence and severity of autoimmune disease [32]. Increased levels of these cytokines in systemic circulation may lead to an increase activity of immune cells and induce tissue damage.

PM2.5 exposure has been associated with elevated levels of circulating monocytes and T cells, but not B cells [33], suggesting activation of T cells via receptors or pathways specific to these immune cells. This is further supported by a study that found that polluted air caused an imbalance of T cells, leading to increased production of proinflammatory cytokines, oxidative stress, and methylation changes [26]. An alternative proposed mechanism of action is that air pollution leads to damaged mitochondria, triggering oxidative stress, which causes an over-production of inflammatory cytokines, and the stimulation of T helper lymphocytes type 1 (Th1) production [26].

Long term exposure to PM2.5 leading to increased cytokine expression has been associated with cardiovascular disease [31], as well as increased incidence of Alzheimer’s disease [34]. Furthermore, *in* *vitro* and *in* *vivo* studies have found that PM induces high levels of several inflammatory markers, including IL-1a, IL-1B, IL-6, IL-8, IL-17, and TNF-α, in the lungs [35, 36]. Another air pollution study linked elevated exposure to NO2 to increased systemic inflammation in COPD patients [37]. Thus, when individuals are exposed to air pollution, there are likely multiple pollutants triggering a range of immune responses simultaneously, activating a variety of pathways that lead to the development of a particular disease. Given this complexity, specific molecular mechanisms are difficult to target clinically, and both fundamental science and drug discovery in this space will rely on improvements in *in* *vivo* and *in* *vitro* models of these diseases.

## Linking Air Pollutants and Bone Health

A number of studies (outlined in Table 1), with increasing pace in the last five years, have shown that in addition to affecting many other physiological systems, a strong link exists between air pollution and bone degeneration. Early indications of a potential relationship between air pollution and bone health arose a 2007 study of Norwegian populations, with an Oslo-based study finding a weak, but still significant, correlation that air pollution was inversely associated with total body BMD [38]. Two additional studies found in 2010 that increased levels of outdoor air pollution could be correlated with loss of bone density and increased rates of forearm fracture [39], and in 2011 that urban women have a 29% higher relative risk of forearm fracture and reduced bone mineral density compared to women in rural areas [40], further hinting that air pollution could affect bone health. Later, in 2015 researchers found similar results in Mexican American populations, reporting a relationship between road traffic metrics, associated ambient air pollution and low BMD [41]. Despite these findings, a systematic review in 2021 found that the links between particulate pollution and osteoporosis are inconclusive, partly due to heterogeneity in study design and subject populations [42].

**Table 1:** The effect of air pollution on bone fracture risk, BMC, BMD, and in vivo bone turnover markers

Authors	Year	Study Type	Sample (n)	Findings
Alvaer et al., *Osteopor Int* [38]	2007	Epidemiological	1525 (men)	PM10, PM2.5 & NO2 linked to lower whole-body BMD
Alver et al., *Osteopor Int* [39]	2010	Epidemiological	1039 (mixed)	PM10, PM2.5 & NO2 linked to forearm fracture risk
Omsland et al., *J Bon Min Res* [40]	2011	Epidemiological	7333 (women)	Increased forearm fracture risk in urban populations
Chen et al., *Osteopor Int* [41]	2015	Epidemiological	1175 (mixed)	Total and pelvic BMD decreased with proximity to heavy road traffic
Prada et al., *Lancet Planet Health* [43]	2017	Epidemiological	9.2 million (women), 1219 (men)	Greater risk of osteoporotic fracture at multiple anatomical sites in areas with higher PM2.5 or black carbon
Mazzucchelli et al., *Osteopor Int* [44]	2018	Epidemiological	4271 (mixed)	SO2, NO & NO2 linked to increased prevalence of hip fractures
Kheirouri et al., *Envir Health Toxicol* [45]	2020	*in* *vivo*	32 (rats)	SO2, O3 & PM did not alter ALP, OC, OPG & PTH in blood samples
Ranzani et al., *Environ Health* [46]	2020	Epidemiological	3717 (mixed)	PM2.5 associated with lower BMC in the spine and hip
Qiao et al., *Environ Res* [47]	2020	Epidemiological	8033 (mixed)	PM1, PM2.5, PM10 & NO2 all linked to increased osteoporosis risk
Adami et al., *Osteopor Int* [48]	2021	Epidemiological	59,950 (women)	PM10 & PM2.5 linked to higher risk of osteoporotic T-score at any site
Prada et al., *Lancet eClinicalMedicine* [49]	2023	Epidemiological	161,808 (women)	PM10, NO, NO2, & SO2 all linked to lower BMD
Qi et al., *J Bon Min Res* [50]	2023	Epidemiological	446,395 (mixed)	PM10, PM2.5, NO2 & NOx all linked to higher fracture risk
Yu et al., *Front Pub Health* [51]	2023	Epidemiological	430,120 (mixed)	PM10, PM2.5, NO2 & NOx interact with genetics to increase fracture risk
Yang et al., *Chemosphere* [52]	2023	Epidemiological	341,311 (mixed)	PM10, PM2.5, NO2 & NOx all linked to lower BMD
Ge et al.,* Environ* *Health Perspec* [53]	2023	Epidemiological *in* *vivo* *in* *vitro*	67,206(mixed) 12 (mice) 4 (replicates)	PM2.5 linked to lower BMD, increased osteoclasts and osteoclastic signalling both *in* *vivo* and *in* *vitro*
Zhang et al., *Arch Osteopor* [54]	2023	Epidemiological	5044 (mixed)	PM10, PM2.5, & NO2 linked to higher osteoporotic fracture risk
Jiang et al., *Arch Med Sci* [55]	2024	Epidemiological	423,796 (mixed)	PM10, PM2.5, NO & NO2 linked lower BMD

(ALP – alkaline phosphatase, OC – osteocalcin, OPG – osteoprotegrin, PTH – parathyroid hormone)

**Fig. 1:**
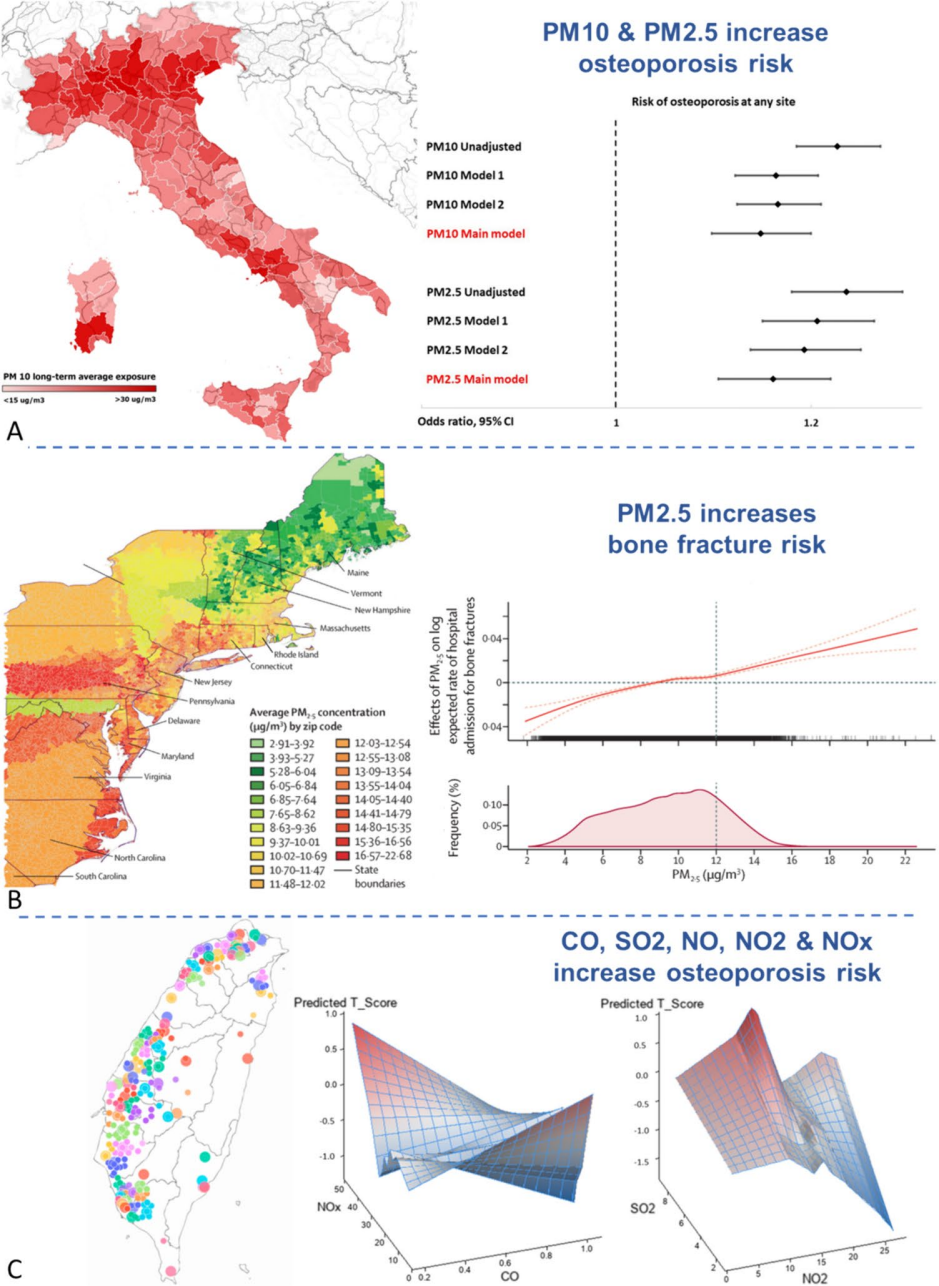
**A** Long-term exposure to PM10 in Italy (2013–2019 average concentration μg/m^3^)[48]. Risk of osteoporosis at any site in patients chronically exposed to PM10 > 30 μg/m^3^ and PM2.5 > 25 μg/m^3^. Model 1 adjusted for age, body mass index (BMI), presence of prevalent fragility fractures, family history of osteoporosis, and menopause. Model 2 adjusted for age, BMI, presence of prevalent fragility fractures, family history of osteoporosis, menopause, glucocorticoid treatment, and comorbidities. Model 3 (main model) adjusted for age, BMI, presence of prevalent fragility fractures, family history of osteoporosis, menopause, glucocorticoid treatment, comorbidities, and macro-area of residency (categorized as northern Italy, central Italy, and southern Italy). **B** Average PM2.5 concentrations per zipcode in the US Northeast between 2003 and 2010[43]. Spline shown for the multivariable-adjusted association between PM2·5 exposure and number of hospital admissions of Medicare enrollees per zipcode, from 2003 to 2010. Horizontal dotted line represents zero effect. **C** Example of nearest neighbour interpolation between measurements in Taiwan [58], with big circles standing for monitoring station and small ones for participants. A synergistic effect of CO and NOx on BMD T-score was found to be statistically significant (*p* = 0.001), as was a synergistic effect between SO2 and NO2 (*p* = 0.004)

It has been shown that short-term air pollution exposure increases hip fracture risk in multiple European populations (Fig. 1A) [44, 48]. Similar associations have also been found in multiple human studies across a wide range of countries in Asia [46, 47, 56–58].

Perhaps most importantly, a recent landmark paper has prospectively determined the impact of criteria air pollutants and their mixtures on BMD in ~ 161,000 postmenopausal women in the US [49], using two separate epidemiological studies to reveal a correlation between air pollution and a ninefold increase in risk of osteoporosis (Fig. 1B) and with general bone damage [43]. This study demonstrated for the first time that from air pollution mixtures, nitrogen oxides likely contribute the most to bone damage and that the lumbar spine is one of the most susceptible sites [43]. Results from these analyses indicated that poor air quality was a possible risk factor for BMD loss and fractures in older individuals and that per each 4.18 μg/m^3^ increase in PM2.5, there is a 4.1% higher rate of hospital admission for bone fractures in older individuals [43, 49]. Thus, in studies using very large population sizes, there now appears to be a clear and significant link between air pollutants and bone health, but the potential underlying mechanism is as yet undiscovered.

This Lancet study by Prada et al. [43] was quickly followed by a flurry of epidemiological studies demonstrating the same effect in other countries [59], including a number leveraging the unique dataset held within the UK Biobank [50–53]. For example, recent reports from Zhang et al. suggested that long-term exposure to PM2.5 was associated with decreased BMD T-score and increased osteoporosis risk among participants from rural areas of China [54]. The UK Biobank studies in particular found clear links between a range of air pollutants and decreased bone mass, decreased BMD and increased risk of fracture within the UK population [50–52], particularly identifying PM2.5 and nitrogen oxides as likely culprit pollutants. A recent additional study applied Mendelian randomisation on UK biobank data, which employed statistical analysis to develop greater confidence in causal links between variables, finding robust stastical evidence affirming a causal relationship between decrease in BMD and increased PM2.5, PM10, NO and NO2 exposure [55]. A number of putative mechanisms have been proposed, all of which generally involve inflammatory signalling [60, 61]; 1) low-grade systemic inflammation affecting osteoblast and osteoclast differentiation and function; 2) oxidative damage in the airway and bone cells from compounds such as heavy metals; 3) endocrine disruption when binding to the receptors in bone cell; and 4) directly or indirectly inducing vitamin D deficiency. However, at present, the specific inflammatory mechanism that causes osteoporosis remains unknown.

Inflammation influences various important signalling pathways in bone health; the release of pro-inflammatory cytokines has been reported to inhibit osteoblast mitogen-activated protein kinases (MAPK) [62] and the WNT–Frizzled–β-catenin pathway [63, 64] that ultimately suppresses the differentiation and activation of osteoblasts. In osteoclasts, activation via inflammatory mechanisms have been shown to amplify osteoclastogenesis, resulting in local bone loss [65].

Previous research into the effect of inflammation on primary cilia showed that cilium length was elongated following IL-1β exposure [66]. Primary cilia mediate a number of key inflammatory pathways in osteocytes [67], and have been shown to play a role in downstream inflammatory signalling [68], increasing the release of inflammatory mediators within bone, and potentially altering the cells’ functional mechanosensation. Similarly, in the context of breast and bone cancer, the osteocyte primary cilium has been shown to mediate TGF-β and TNF-α inflammatory signalling in the metastatic niche [69], highlighting this organelle as a potential target for air-pollution mediated inflammation.

Air pollution-induced osteoporosis is therefore a significant challenge for health systems, as the global population is rapidly ageing and mortality increases substantially in elderly patients in the years after a hip or vertebral fracture. Most importantly, the global population is increasingly urban and exposed to these pollutants, with the UN predicting 68% of the global population residing in cities by 2050 [70]. Demonstrating the importance of place, specific localities and social groups are exposed to poorer air quality and therefore higher risk of bone degeneration.

A key challenge to identifying the molecular mechanism underlying these destructive relationships, as demonstrated by the few animal studies on the topic [45, 53], is that rodent models do not age or remodel bone in the same manner as humans, and do not naturally develop osteoporosis. This is especially true given that the mechanisms likely involve lung-immune-bone crosstalk, and rodents have been shown to have vastly different immune and healing responses to humans [71]. Indeed, the first animal study carried out found contradictory interactions, with little indication of bone damage in a rat model resulting from air pollutants and increased blood levels of vitamin D due to exposure to some air pollutants [45]. The only other animal study to date, performed on male C57BL/6 mice, did indeed find that PM2.5 exposure resulted in increased osteoclastogenesis, dysregulated osteogenesis and shortened femur length, although no significant differences in femur structure or BMD were detected [53]. This study did also conduct a simple *in* *vitro* experiment, in which they found that osteoclastogenic behaviour and signalling was disrupted by conditioned media from macrophages exposed to PM2.5 [53]. Taken together, these limited experiments suggest that further investigation to unpick these molecular mechanisms is likely to require sufficiently complex human-derived *in* *vitro* models that can include components of the immune system (e.g. organ-on-a-chip or microphysiological systems) [72, 73]. Indeed, guidance from regulatory agencies (e.g. FDA, EMA) and funding bodies (e.g. NIH, Horizon Europe) worldwide has been updated in the past five years to encourage the development of more accurate in vitro models, including to address conditions with complex immune involvement as may occur in pollution-related skeletal degeneration.

Considering the expanding body of evidence implicating the effects of air pollution on various organ systems, paired with the research into inflammation leading to loss of BMD and increased fracture risks, it logically follows that air pollution triggers an inflammatory response in bones, leading to degeneration and diseases like osteoporosis. As there has been little research to study the effect air pollution has on bone health, the precise mechanisms are currently unknown.

## Conclusions

Research over the past five years has established a link between air pollution and bone degeneration, and an association with an increased fracture risk. Public interest in this challenge recently highlighted in an article in Science [74]. Increased risk of osteoporosis has been specifically identified, implying that systemic inflammatory factors may induce early onset of osteoporosis. Mounting evidence appears to identify nitrogen oxides and PM2.5 as irritants of key interest. However, while major steps have been taken in understanding the epidemiological and population-level associations, the precise mechanisms through which these pollutants induce bone damage or instigate osteoporotic cascades remain to be elucidated. Further study is required to identify the impact of different types of pollutants, the resulting impact of inflammation on bone health and the underlying biological pathways. Given the deficiencies of animal models of air pollution and bone diseases, it is clear that new complex human *in* *vitro* models such as organ-on-a-chip technology will be required in this field.

## Key References

- Prada, D.; Zhong, J.; Colicino, E.; Zanobetti, A.; Schwartz, J.; Dagincourt, N.; Fang, S.C.; Kloog, I.; Zmuda, J.M.; Holick, M. Association of air particulate pollution with bone loss over time and bone fracture risk: analysis of data from two independent studies. Lancet Planet. Heal. 2017, 1, e337–e347.
  - This paper used extremely large datasets in multiple cohorts to establish strong statistical evidence that poor air quality is a modifiable risk factor for bone fractures and osteoporosis, especially in low-income communities.
- Kheirouri, S.; Alizadeh, M.; Abad, R.M.S.; Barkabi-Zanjani, S.; Mesgari-Abbasi, M. Effects of sulfur dioxide, ozone, and ambient air pollution on bone metabolism related biochemical parameters in a rat model. Environ. Anal. Heal. Toxicol. 2020, 35.
  - This study represents the first *in* *vivo* experiment to investigate the link between air pollutants and bone health, with contradicting findings suggesting that more complex *in* *vitro* models are required to establish an underpinning mechanism.
- Ge Q, Yang S, Qian Y, Chen J, Yuan W, Li S, Wang P, Li R, Zhang L, Chen G, Kan H. Ambient PM 2.5 Exposure and Bone Homeostasis: Analysis of UK Biobank Data and Experimental Studies in Mice and in Vitro. Environmental Health Perspectives. 2023 Oct 4;131(10):107002.
  - This study conducts both *in* *vivo* mouse and *in* *vitro* conditioned media experiment to investigate the effects of PM2.5 pollutants, finding disruption to osteoclastogenisis and osteoclastic signalling in both models. However, neither finds strong indications of loss in bone mineral, further suggesting that more complex *in* *vitro* models are required.
- Yu, X.-H.; Cao, H.-W.; Bo, L.; Lei, S.-F.; Deng, F.-Y. Air pollution, genetic factors and the risk of osteoporosis: A prospective study in the UK biobank. Front. Public Heal. 2023, 11, 1119774.
  - This study applied the resources of the UK Biobank to find that that exposure to various air pollutants, individually or jointly, could improve the risk of developing OP and fractures, and increased the risk by interacting with genetic factors.
- Jiang R, Qu Q, Wang Z, Luo F, Mou S. Association between air pollution and bone mineral density: a Mendelian randomization study. Archives of Medical Science. 2024.
  - This study further leveraged the data held within the UK biobank, applying a Mendelian randomisation method to give statistical confidence of a robust causal link between lower BMD and exposure to nitrogen oxides and particulate matter.

## Data Availability

Data presented and discussed in this review is available at source in the relevant referenced studies.
